# Accelerometery vs. video-derived stroke parameters in high-level swimmers

**DOI:** 10.17159/2078-516X/2021/v33i1a9483

**Published:** 2021-04-21

**Authors:** C Musson, M Kramer

**Affiliations:** 1 Human Movement Science Department, Nelson Mandela University, Gqeberha, South Africa; 2 Physical Activity, Sport and Recreation (PhaSRec) Unit, Human Movement Sciences Department, North-West University, Potchefstroom, South Africa

**Keywords:** accelerometers, elite swimmers, kinematics, video analysis

## Abstract

**Background:**

Swimming is a multifaceted sport with nuanced performance parameters that tend to vary according to the swimmer’s stroke style. The extraction and analyses of swim parameters, such as lap time (LT), stroke length (SL), stroke rate (SR) and velocity are time-consuming. This may be eased and to some extent automated by the use of cost-effective tri-axial accelerometers.

**Objectives:**

To determine the validity of tri-axial accelerometers across all four stroke styles, and to investigate kinematic differences in stroke styles using accelerometer-based data.

**Methods:**

Twelve elite swimmers were recruited for the study. The group consisted of five male (age: 22.2 ± 2.6 years; height: 1.84 ± 0.08 m; weight: 76.2 ± 3.6 kg) and seven female (age: 20.7 ± 2.1 years; height: 1.68 ± 0.08 cm; weight: 62.0 ± 6.3 kg) swimmers.

**Results:**

There was a small but significant bias for accelerometery data compared to video data across most parameters and stroke styles except for stroke length and stroke count (p > 0.05). However, accelerometery-derived SR, SL and velocity can be considered practically useful based on the training requirements of coaches. Parameters derived from video analysis compared to accelerometery were highly correlated (r > 0.91) and therefore consistent regardless of the method of analysis.

**Conclusion:**

Although slight differences were present between the video and accelerometer data, these differences were not practically meaningful.

Optimal performance across sporting codes is typically based on the careful interplay between the athlete’s biomechanical, physiological and psychological factors on the one hand, and economic and social factors on the other.^[1,2]^ For a bilateral and cyclical sport such as swimming, the most important parameter governing performance would be the optimisation of swimming velocity to complete a given race distance in the fastest time possible.^[1]^ Although the factors affecting swimming velocity are multifactorial (e.g. energetics, index of coordination, arm span, motivation, etc.), the maximisation of swim velocity is mostly dependent on the relationship between stroke length (SL) and stroke rate (SR).^[1,3,4]^ Both SL and SR are highly variable and are, in part, contingent on the stroke style utilised, namely freestyle, breaststroke, backstroke or butterfly.^[3]^ It is traditionally the role of the coaches to keep a careful account of each of the parameters mentioned to improve the performance of their swimmers more effectively. For most coaches such a task is increasingly difficult and can often be imprecise due to the technical aspects that must be accounted for. The difficulty of the task may be compounded by the number of athletes each coach must deal with at any time.^[5]^

The aforementioned challenges have created opportunities for the use of alternative technologies which may aid coaches in their tasks. Such technologies include heart rate monitors, inertial measurement units, and accelerometers which can keep account of factors such as physiological effort and various swim kinematics.^[6]^ Accelerometers in particular have proven to be beneficial in swimming stroke and turning analyses, automatic stroke phase recognition^[7]^ and performance feature extractions (e.g. stroke length).^[8]^ Therefore the use of accelerometery appears to be a viable and potentially low-cost option for providing coaches with technical support. Despite this, there is currently limited research on: (i) the validity of accelerometers across different accelerometer types, and (ii) the utility of accelerometers to differentiate different stroke styles in high-level swimmers.

The objectives of the present study were therefore two-fold: (i) to determine the validity of parameters derived from a commercially available accelerometer compared to parameters derived from video analysis, and (ii) to identify kinematic differences in stroke styles using accelerometer-derived data in high-level swimmers.

## Methods

### Study design

This study used a one-group posttest-only design. The rationale for such a design was that the principle parameters of interest were related to the validity of the accelerometers across different swim strokes and swim distances.

### Participants

A total of 12 South African national level swimmers from different swim clubs volunteered for this study. To be included, swimmers must have represented at a national level (e.g. Junior Nationals, Youth Nationals, Youth Elite or Senior National level), and have been free of injury prior to testing. Based on the latter criterion, the sample group was comprised of five male (age: 22.2 ± 2.6 years; height: 1.84 ± 0.08 m; weight: 76.2 ± 3.6 kg; national swim experience: 6.1 ± 3.0 years) and seven female (age: 20.7 ± 2.1 years; height: 1.68 ± 0.08 cm; weight: 62.0 ± 6.3 kg; national swim experience: 7.1 ± 2.5 years) swimmers. This study was conducted according to the guidelines of the Declaration of Helsinki and all procedures were approved by the institutional Research Ethics Committee (H18-HEA-HMS-007). All swimmers were required to provide written informed consent after having received all necessary instructions related to the study. Instructions to participants included: (i) no consumption of alcohol or caffeine 48 hours prior to testing, (ii) avoiding strenuous exercise 48 hours prior to testing, and (iii) arriving for testing well hydrated, but two to three hours postprandial.

### Data collection

Participants visited the testing facility on two separate occasions during their pre-season training period. The first visit was for completing informed consent forms and familiarisation with the equipment setup. The second visit, separated from the first by 24–48 hours was used for recording: (i) anthropometric data, such as height (m), weight (kg) and arm span (m), and (ii) the swim assessment which consisted of a 4 x 50 m individual medley (IM) completed in a 25 m indoor pool (water temperature: 25–27°C). The IM consists of the consecutive completion of all four swimming strokes in a pre-specified order, namely butterfly, backstroke, breaststroke and freestyle. Prior to testing, participants were fitted with two tri-axial accelerometers (GeneActiv, ActiveInsights, UK; sampling at 100 Hz) worn on the left wrist and strapped to the upper back of the torso, as well as a heart rate monitor (Polar H7, Kempele, Finland). Once fitted, participants were instructed to complete a self-paced warm-up lasting five minutes, followed by a five minute cooldown period to have physiological values return to baseline. For the IM, all participants completed all four strokes, regardless of stroke specialisation. Participants were also instructed to swim at a slow to medium pace as they were video recorded with a custom-made two-camera-trolley system that allowed for video recording from below (GoPro 4, GoPro Inc., San Mateo, California, United States) and perpendicular to (Sony Cyber-shot DSC-RX10 MK III, Sony Electronics Inc., New York, United States) the line of travel. Both video cameras recorded at 100 Hz which allowed for 1:1 synchronisation with the tri-axial accelerometers.

### Parameter calculations

**Fig. 1:**
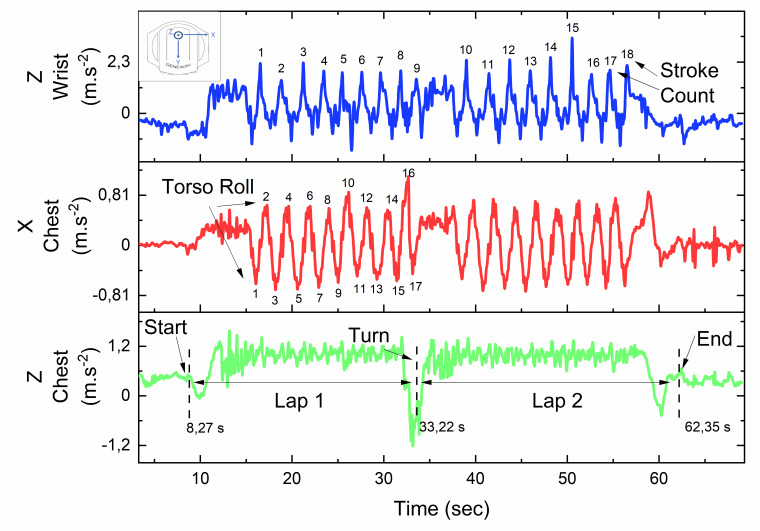
Tri-axial accelerometer data from the wrist and trunk of a representative athlete for the 50 m (2 x 25 m) backstroke component during the individual medley (IM).

Accelerometer data were downloaded and exported for analysis in OriginPro (OriginLab, version 2019b, USA), where data were filtered using a low-pass fourth order, zero-lag Butterworth filter with a cut-off frequency of four-six Hz. Depending on the stroke, two different axes were used to extract the kinematic parameters, dictated by the primary plane of movement used by the swimmer during the execution of the stroke. For the freestyle and backstroke, the z-axis from the wrist accelerometer provided information related to stroke count, whereas the x- and z-axes from the trunk accelerometer provided information related to lap times (see Fig. 1). Conversely, for the breaststroke and butterfly, the y-axis from the wrist accelerometer provided information related to stroke count, whereas the x- and y-axes provided information related to lap times.

For video data, the completed lap times were recorded as the duration from the start signal to when the hand touched the wall at the end of the final stroke. For accelerometer data, the completed lap times were derived from the y-axis of the chest unit, where there was a deviation away from 1g (start), and when the acceleration would return to 1g; which would occur when the torso was upright (see green plot in Fig. 1). The relevant peaks and troughs from the accelerometers were analysed using specialised peak-finding algorithms (OriginPro, OriginLab, version 2019b, USA) which could then be used to calculate parameters such as SR, average SL and average swim velocity. Comparisons between the video- and accelerometer data could then be made. Parameters were calculated using the following equations which would be applied to both video- and accelerometer-derived parameters:


[1] Average swim velocity (m/s)=distance (m)/lap time (s)

[2] Average stroke rate (stroke/s)= number of strokes (n)/lap time (s)

[3] Stroke length (m/stroke)=average swim velocity (m/s)/SR  (stroke/s)

### Statistical analysis

All data are presented as means ± standard deviation (SD) unless otherwise stated. Data were assessed for normality using the Shapiro-Wilk test with the level of significance set at 0.05. For the first objective, a Bland-Altman analysis was performed to determine the bias and limits of agreement (LA), with 95% confidence intervals (CI_95%_), between the video (criterion) and accelerometer (alternative) parameters (i.e. lap time, SL, SR, velocity and stroke count). A correlation analysis was used to show the magnitude and direction between accelerometer- and video-derived parameters and the relationship between these parameters where the absolute value of the correlation coefficient was interpreted as follows: trivial: < 0.10, small: < 0.30, moderate: < 0.50, large: < 0.70, very large: < 0.90, almost perfect: ≤ 1.00.^[9]^ For the second objective, the two one-sided t-test (TOST) analysis for paired data was used to assess whether differences between accelerometer-derived vs. video-derived parameters were statistically equivalent and therefore practically meaningful. Equivalence bounds were based on raw scores, with uncertainty in the point estimates for the mean difference being presented as 90% confidence intervals (two-sided test). ^[10]^ Through careful discussion with coaches, the equivalence bounds were set at a given deviation from zero: velocity = *±* 0.10 m.s^−1^ (i.e. within ~ 0.5 km.hr^−1^), SR = *±* 0.04 strokes.s^−1^ (i.e. within ~ 2–3 strokes.min^−1^ [or ~ 0.03–0.05 strokes.s^−1^]), SL = *±* 0.02 m.stroke^−1^ (~ 20 cm.stroke^−1^, being essentially equivalent to ~ 1 hand span), LT = *±* 1.50 s. The statistical analyses were completed using Microsoft Excel (Microsoft Corporation, USA) and OriginPro (OriginLab, version 2019b, USA).

## Results

The summary statistics (M ± SD) of key swim parameters for each of the four different stroke styles derived from both accelerometers and video analysis are presented in Table 1.

**Fig. 2:**
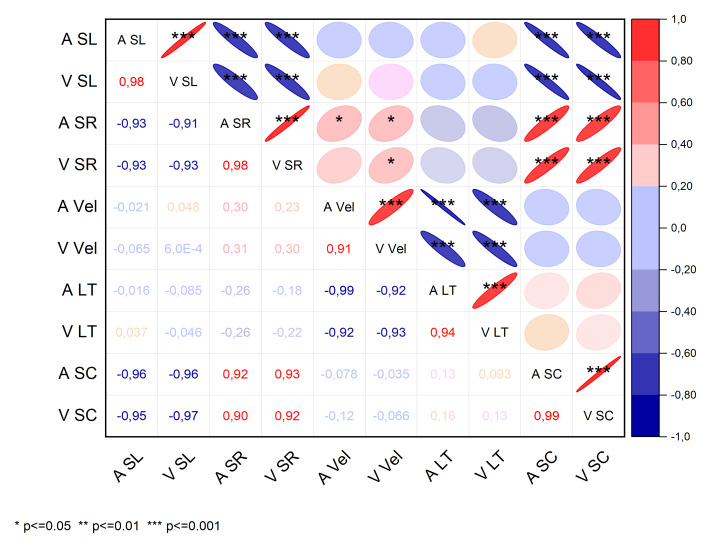
Correlation matrix of kinematic parameters across all stroke styles. A, accelerometer; V, video; SL, stroke length; SR, stroke rate; vel, velocity; LT, lap time, SC, stroke count. Upper triangular portion shows the confidence magnitude and direction of the relationship (* denotes statistical significance). Lower triangular portion shows the correlation coefficients.

The correlations between accelerometer-derived and video-derived parameters are shown in Figure 2. The parameters of interest were pooled for all stroke styles to provide an overview of the magnitude and direction of the relationship between accelerometer and video data.

Irrespective of stroke style, there was evidence of statistically significant bias (i.e. mean difference away from zero) between accelerometer- vs. video-derived parameters for average swim velocity (n=48, bias = −0.05 m.s^−1^, CI_95_ [−0.06, −0.04], p < 0.001), *SR* (n=48, bias = −0.02 str.s^−1^, CI_95_ [−0.02, −0.01], p < 0.001), and *LT* (n=48, bias = −1.38 s, CI_95_ [1.16, 1.60], p < 0.001) (see Table 2 for individual stroke style data). Across all styles, no statistically significant differences were evident for SL (n=48, bias = 0.00 m.str^−1^, CI_95_ [−0.03, 0.04], p = 0.795) or stroke count (n=48, bias = 0.10, CI_95_ [−0.09, 0.30], p = 0.280). The discrepancies between video- and accelerometer data for the first three parameters are thought to be due to errors from the accelerometer data with regards to the identification of the turning points for each lap within the IM. Such errors would affect the calculated lap durations which in turn would affect calculations such as the average swim velocity and SR.

**Fig. 3:**
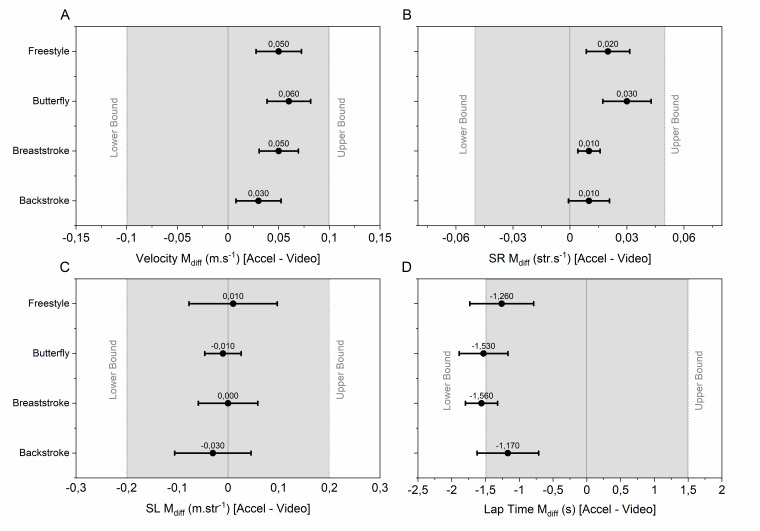
TOST results showing the mean difference (M_diff_) with CI_90_ error bars between accelerometer and video parameters for velocity (panel A), stroke rate (SR: panel B), stroke length (SL: panel C) and lap time (panel D). The predetermined region of equivalence is shown in grey, with the lower and upper bounds being shown by dotted vertical lines. The zero line is shown by the solid vertical line.

Based on the equivalence bounds set by the coaches, it is clear that the differences between video- and accelerometer parameters fell well within the equivalence bounds for swimming velocity, SR and SL (see Fig. 3, panels A-C). The LT however would be significantly over-predicted by the accelerometers compared to the video data (see panel D, Fig. 3). Since accurate accelerometer-based LT identification was based on an upright torso at the end of a lap, any delay in getting the torso upright would lead to an overestimation in LT compared to video analyses.

## Discussion

The main finding of the present study was that although the accelerometer-derived parameters showed statistically significant bias compared to video-derived parameters, the primary variables of interest (i.e. SL, SR, and velocity) were well within the confidence bounds of what would be considered practically meaningful. Furthermore, the authors have provided an overview of the kinematic parameters of all four stroke styles for high-level swimmers and how these compare when derived from either accelerometer- or video-based analyses.

Although statistically significant, bias between video- and accelerometer data were present, implying that the accelerometers may not be considered a ‘true’ surrogate for video data. The data derived from these units are still considered practically useful for coaching and training purposes. The accelerometers used in the present study also show potential utility for monitoring swimming performance for multiple athletes during training across all stroke styles. While certain commercially available activity monitors include the capability to detect stroke events, these are typically limited to only a few strokes and show similar errors to those observed in the present study^[11]^, thereby potentially motivating the use for more specialised accelerometery devices.

The automated monitoring of swim training is considered an important component of performance improvement. As the training season progresses, swimmers usually experience changes in stroke length, enhanced stroke efficiency, as well as decreased lap times.^[3]^ Fluctuations in key performance parameters would require the regular, repeated monitoring and analysis by coaches, which would be considerably more difficult and time-consuming with larger athlete cohorts.^[12]^ Video analysis is typically an attractive option; however, a key disadvantage of quantitative video analysis relates to the time taken to manually digitise footage (e.g. 7–27 hours depending on athlete count and analysis complexity), which is often coupled with a higher probability for introducing human error.^[12,14]^ Although automation of the digitisation process is continuously evolving, it requires the procurement of multiple cameras, sophisticated software and placement of key anatomical markers, the combination of which would disproportionately increase costs, decrease viability and may inadvertently affect the swimmers’ kinematics.^[12]^ The use of affordable technologies, such as tri-axial accelerometers (~R 3000), may be a considerable benefit in monitoring training progression that would also substantially decrease analysis time and enhance the reliability of athlete feedback.^[5,12,13]^ The present study has shown that accelerometer data is not only a viable alternative, but also highly correlated with video data (r >0.91). The accuracy of the results obtained here are on a par with those of other studies, even though the sample investigated was different.^[12,13]^ Furthermore, key kinematic parameters evaluated in the present study, such as swim velocity, SR, and SL, are practically equivalent between both methods of analysis which enhances the real-world utility of such devices. Although accelerometery undoubtedly shows promise as a measurement and analysis tool, certain factors, such as qualitative technical aspects, cannot yet be derived from accelerometery alone. Until such a time that the nuanced details of the swim stroke can be parameterised with accelerometery using more powerful and versatile algorithms, it is highly probable that video analysis, although cumbersome, will continue to remain a central component of a holistic swimming analysis.

A logical next step would be to evaluate: (i) whether the accelerometers and algorithms used in the present would apply beyond the distances and athletic cohort used in the present study, (ii) differences across different swimming speed profiles (i.e. long, slow distance vs. sprint swimming), and (iii) differences in stroke technique within a given swim stroke. The evaluation of longer distances is primarily limited by the video technology as this would need to be available for data validation. ^[6,14]^ In their study, Ganzevles et al.^[13]^ found that the 1200 m distance yielded reliable and accurate results although the accurate detection of finishing time was still problematic. Similarly, changes in the acceleration profile of the wrist, considered a proxy for swimming intensity, would change the parameter extraction and may therefore change the accuracy of the algorithm for detecting key aspects of a stroke.^[6]^ In the present study a slow stroke rate was purposefully utilised due to the complexities of the data collection process. Ganzevles et al.^[13]^; however, noted that higher stroke rates tend to lead to a greater spread in the accelerometer data which may be attributed to a greater number of irregularities in the stroke rhythm. Finally, although each stroke exhibits similarities in the acceleration profile across athletes, it is important to note that athletes do exhibit subtle differences in stroke phase durations and hand/wrist placement during a given stroke.^[15]^ Such differences may introduce subtle nuances in the acceleration profile that may affect the accuracy of timing information derived from accelerometers. Future research should therefore focus on specific feature extraction within the acceleration profile and track this across a swimming season and see whether the acceleration profile is sensitive to the effects of an intervention.

### Limitations

Several limitations were present in this study. The overall swim velocity was restricted to ensure that two-dimensional video data could adequately capture the full stroke profile of the swimmer. Longer swim distances were omitted as the validity of the accelerometers across stroke styles was unknown; therefore, whether the data transfers to longer swim distances is presently not verified. The sample used in the present study was homogenous (i.e. elite), therefore whether kinematic parameters can be easily identified for non-elite and/or recreational swimmers is presently unknown on the basis that stroke kinematics may be more varied.

Future research should investigate whether accelerometery could be used to extract more nuanced features related to stroke mechanics, such as in-sweep, out-sweep and recovery times to provide even more useful information for the coach.

A further limitation relates to the fact that these authors generalised the equivalence bounds across all swim strokes to assess the practical utility of the accelerometers as a whole; and they do acknowledge that the bounds for each parameter may change depending on the stroke style and this should be considered in future. Finally, all analyses in the present study were post hoc, and therefore future research should investigate whether instantaneous data streaming would be possible, as this may be more meaningful for the coach and/or athlete by providing immediate, rather than delayed, feedback.

## Conclusion

Accelerometers are a meaningful tool for monitoring swimming kinematics across different stroke styles. Although slight differences were present between video and accelerometer data, they were not practically meaningful. The accelerometers used in the present study therefore show promise as a training monitoring tool on the basis that they are affordable, show high correlation and practical equivalence for key parameters, such as swimming velocity, SR, SL and SC.

## Figures and Tables

**Table 1:** Descriptive statistics of accelerometer-derived and video-derived kinematic parameters for all swim strokes

Parameter	Freestyle		Butterfly		Breaststroke		Backstroke	
	Accel	Video	Accel	Video	Accel	Video	Accel	Video

**Stroke count (n)**	18.17 ± 3.9	18.00 ± 3.93	22.75 ± 4.69	22.92 ± 4.46	19.50 ± 5.30	19.17 ± 5.67	16.92 ± 3.12	16.83 ± 2.76
**Stroke length (m.stroke** **^−1^** **)**	2.89 ± 0.60	2.90 ± 0.58	2.30 ± 0.49	2.29 ± 0.48	2.78 ± 0.78	2.78 ± 0.80	3.09 ± 0.58	3.06 ± 0.48
**Stroke rate (stroke.s** **^−1^** **)**	0.37 ± 0.09	0.39 ± 0.09	0.47 ± 0.10	0.50 ± 0.10	0.37 ± 0.08	0.38 ± 0.09	0.33 ± 0.07	0.34 ± 0.06
**Velocity (m.s** **^−1^** **)**	1.03 ± 0.07	1.08 ± 0.07	1.04 ± 0.09	1.10 ± 0.10	0.96 ± 0.08	1.01 ± 0.09	0.99 ± 0.08	1.02 ± 0.10
**Lap time (s/25 m)**	24.44 ± 1.56	23.18 ± 1.46	24.29 ± 2.22	22.76 ± 2.05	26.40 ± 2.30	24.84 ± 2.23	25.46 ± 1.92	24.29 ± 2.12

Data are expressed as mean ± SD. Accel, accelerometer.

**Table 2:** Summary of Bland-Altman statistics between the accelerometer- and video-derived kinematic parameters for all stroke styles

Parameter	Bias Estimate [CI_95_]	Lower LA [CI_95_]	Upper LA [CI_95_]	p-value
**Freestyle**				
Velocity (m.s^−1^)	−0.05 [−0.07, −0.02]	−0.13 [−0.18, −0.08]	0.04 [−0.01, 0.09]	0.005*
Stroke length (m.stroke^−1^)	−0.01 [−0.12, 0.09]	−0.35 [−0.54, −0.16]	0.32 [0.13, 0.51]	0.778
Stroke rate (strokes.s^−1^)	−0.01 [−0.03, −0.00]	−0.05 [−0.08, −0.03]	0.03 [0.01, 0.05]	0.083
Lap time (s)	1.26 [0.69, 1.84]	−0.51 [−1.51, 0.50]	3.03 [2.02, 4.04]	<0.001*
Stroke count (n)	0.17 [−0.36, 0.70]	−1.47 [−2.40, −0.54]	1.80 [0.87, 2.74]	0.504
**Butterfly**				
Velocity (m.s^−1^)	−0.06 [−0.09, −0.04]	−0.14 [−0.19, −0.10]	0.02 [−0.03, 0.06]	<0.001*
Stroke length (m.stroke^−1^)	0.01 [−0.03, 0.05]	−0.11 [−0.18, −0.04]	0.13 [0.06, 0.19]	0.674
Stroke rate (strokes.s^−1^)	−0.03 [−0.04, −0.01]	−0.07 [−0.10, −0.05]	0.02 [0.01, 0.04]	0.002*
Lap time (s)	1.53 [1.09, 2.00]	0.19 [−0.56, 0.95]	2.86 [2.10, 3.61]	<0.001*
Stroke count (n)	0.17 [−0.41, 0.08]	−0.93 [−1.37, −0.49]	0.60 [0.16, 1.03]	0.166
**Breaststroke**				
Velocity (m.s^−1^)	−0.06 [−0.07, −0.05]	−0.10 [−0.13, −0.08]	−0.02 [−0.04, 0.01]	<0.001*
Stroke length (m.stroke^−1^)	−0.01 [−0.08, 0.07]	−0.24 [−0.38, −0.12]	0.23 [0.10, 0.37]	0.870
Stroke rate (stroke.s^−1^)	−0.02 [−0.03, −0.00]	−0.05 [−0.08, −0.03]	0.02 [0.00, 0.04]	0.018*
Lap time (s)	1.56 [1.28, 1.85]	0.68 [0.18, 1.18]	2.45 [1.94, 2.95]	<0.001*
Stroke count (n)	0.33 [−0.08, 0.75]	−0.94 [−1.67, −0.22]	1.61 [0.88, 2.34]	0.104
**Backstroke**				
Velocity (m.s^−1^)	−0.03 [−0.06, −0.01]	−0.11 [−0.16, −0.67]	0.05 [0.00, 0.09]	0.019*
Stroke length (m.stroke^−1^)	0.03 [−0.06, 0.13]	−0.26 [−0.43, −0.09]	0.33 [0.16, 0.50]	0.458
Stroke rate (strokes.s^−1^)	−0.01 [−0.02, 0.00]	−0.04 [−0.06, −0.03]	0.02 [0.00, 0.04]	0.047*
Lap time (s)	1.17 [0.61, 1.73]	−0.57 [−1.56, 0.42]	2.93 [1.91, 3.89]	<0.001*
Stroke count (n)	0.08 [−0.34, 0.51]	−0.12 [−1.97, −0.48]	1.39 [0.65, 2.14]	0.674

* indicates significance (p<0.05);

CI_95_, 95% confidence interval; LA, limits of agreement.
